# Application of an interspinous process device after minimally invasive lumbar decompression could lead to stress redistribution at the pars interarticularis: a finite element analysis

**DOI:** 10.1186/s12891-019-2565-5

**Published:** 2019-05-15

**Authors:** Hao-Ju Lo, Chen-Sheng Chen, Hung-Ming Chen, Sai-Wei Yang

**Affiliations:** 1 Department of Biomedical Engineering, National Yang-Ming University, No.155, Sec.2, Linong Street, Taipei, 11221 Taiwan; 2 Department of Orthopedic Surgery, Dali Branch, Jen-Ai Hospital, 483 Dong Rong Rd, Dali, Taichung Taiwan; 3 Department of Physical Therapy and Assistive Technology, National Yang-Ming University, No.155, Sec.2, Linong Street, Taipei, 11221 Taiwan; 4 Department of Orthopedic Surgery, Renai Branch, Taipei City Hospital, No. 10, Section 4, Ren’ai Road, Da’an District, Taipei City, 106 Taiwan

**Keywords:** Interspinous process device, Minimally invasive, Finite element model, Pars interarticularis

## Abstract

**Background:**

An interspinous process device, the Device for Intervertebral Assisted Motion (DIAM™) designed to treat lumbar neurogenic disease secondary to the lumbar spinal stenosis, it provides dynamic stabilization after minimally invasive (MI) lumbar decompression. The current study was conducted using an experimentally validated L1-L5 spinal finite element model (FEM) to evaluate the limited decompression on range of motion (ROM) and stress distribution on a neural arch implanted with the DIAM.

**Methods:**

The study simulated bilateral laminotomies with partial discectomy at L3-L4, as well as unilateral and bilateral laminotomies with partial discectomy combined with implementation of the DIAM at L3-L4. The ROM and maximum von Mises stresses in flexion, extension, lateral bending, and axial torsion were analyzed in response to the hybrid protocol in comparison with the intact model.

**Results:**

The investigation revealed that decreased ROM, intradiscal stress, and facet joint force at the implant level, but considerably increased stress at the pars interarticularis were found during flexion and torsion at the L4, as well as during extension, lateral bending, and torsion at the L3, when the DIAM was implanted compared with the defect model.

**Conclusion:**

The results demonstrate that the DIAM may be beneficial in reducing the symptoms of stress-induced low back pain. Nevertheless, the results also suggest that a surgeon should be cognizant of the stress redistribution at the pars interarticularis results from MI decompression plus the application of the interspinous process device.

## Background

Lumbar spinal stenosis (LSS) is a common spinal disorder, in which the bulged intervertebral disc, the hypertrophic facet capsular ligament, as well as ligamentum flavum narrows the spinal cord or root, results in radiculopathy or myelopathy that causes the low-back or leg pain [1]. The optimal surgical treatment for LSS haven’t yet been clearly set, but a bilateral decompression laminectomy to enlarge the spinal canal in order to free the compressed nerves is the typical surgical approach [2]. In general, the dorsal decompression procedure produces instant pain relief and restore the daily functional activities [3].

The construct procedure involves the resections of the lamina as well as partial or total articular process. However, the clinical studies revealed that a potential postoperative iatrogenic spondylolisthesis, which requires further revision [4, 5]. Various minimally invasive (MI) laminotomies have been proposed in order to target the pathologic structures while minimizing segment instability and preserve the maximum spinal bony structure. It is currently a surgical technique commonly used for the treatment of LSS [6, 7]. Studies have shown that there are several advantages of using the MI lumbar decompression which includes the decreasing of blood loss, operative time, duration of hospital stay, rates of infection, and the time required to return to work [8, 9].

However, the MI lumbar decompression has the potential risk of bony fracture of the pars interarticularis due to increasing stresses over the lamina during the procedure [10].

A number of interspinous process devices (IPDs) have been developed in recent years as an alternative to the spinal fusion. It serves not only as a flexible distractor but also functions as a stress absorber in order to provide dynamic stabilization in the treatment of the spinal stenosis [11]. The IPDs are placed over the interspinous processes, and it elevates the foraminal height, offloads the facet joints and the ligamentum flavum, allowing more room for the compressed nerve roots [12, 13]. The Device for Intervertebral Assisted Motion (DIAM™) spinal stabilization system (Medtronic, Ltd., USA), made of silicone rubber with a polyethylene coat, provides aforementioned spinal implant functions with a profound clinical satisfaction in lumbar spinal disorder related neuropathology treatment [14]. However, spinal stenosis is usually accompanied by a bulged-herniated disc, and a discectomy is often performed as part of the decompression process. Few previous studies have offered any insight into the relationships between the posterior elements in terms of the biomechanical effects of surgeries involving DIAM implantation. It is thus still debatable whether added instrumentation is beneficial and whether the DIAM can provide greater stability than decompression alone.

The design and efficacy of orthopaedic implants can be validated by either in vitro or in vivo experiments, and the computational biomechanical analysis provides the product design verification as well as the potential implanted system failure prediction. Several finite element modelling of the lumbar spinal column have been intensively reported. For instance: the effect of different MI approaches without implants [1, 10], the stress distribution over the interarticular region due to lumbar spondylolysis [15], the stabilized effect of IPD positioning [16], the range of motion of the functional unit before and after the IPD implanted [17], the effect of injured disc model with/o IPD instrumented on the ROM and stress at the disc annulus/implant at the index as well as the superior adjacent levels under a loading control protocol [18], and IPD implanted model under a hybrid protocol [19]. The hybrid protocol is more anatomically relevant, which provides more realistic spinal motion and loads in the computational analysis after surgical procedures and implantation [20].

Therefore, the purpose of this study attempted to evaluate the biomechanical effect of DIAM IPD in the region of the pars interarticularis, the adjacent and index segments by using the hybrid protocol in the ligamentous lumbar spine FE model, which has rarely been applied in the computational spine research. A clinical MI approach of laminotomies and discectomy were conducted in this study which provided a mimics reality surgical condition. To our knowledge, such laminotomies and discectomy MI models combined with DIAM implantation under the hybrid protocol has not been investigated.

## Methods

### FEMs of the lumbar spine and implant

**Fig. 1:**
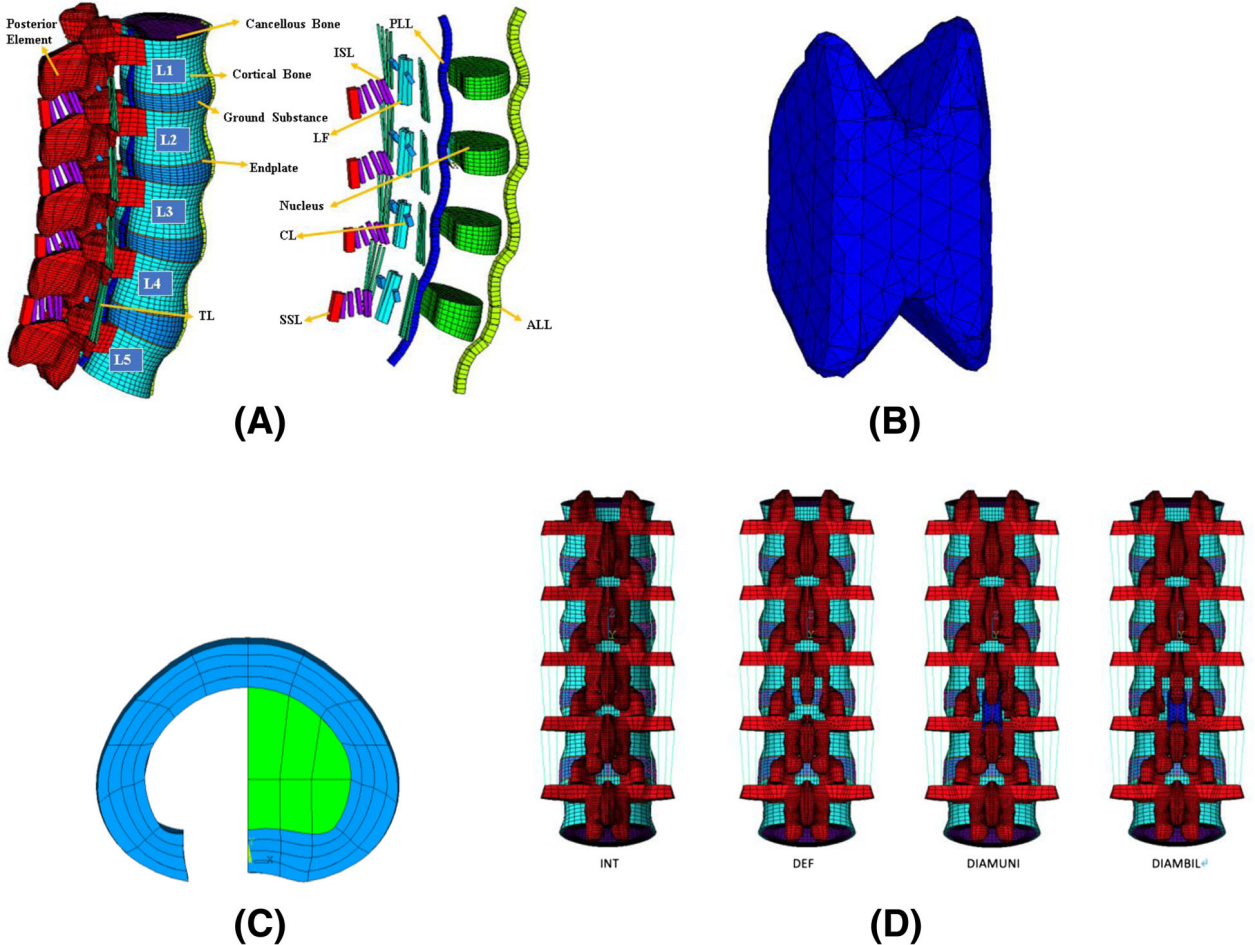
Spine and implant FEMs used in this study. **a** The osseous structures, intervertebral discs, and ligaments of the intact spine. **b** The DIAM. **c** At the L3-L4 disc space, partial discectomy was performed. **d** The four FEMs used in this study. ALL = anterior longitudinal ligament; CL = capsular ligament; ISL = interspinous ligament; LF = ligamentum flavum; PLL = posterior longitudinal ligament; SSL = supraspinous ligament; TL = transverse ligament

The 3-dimensional FE model of the ligamentous lumbar spine L1-L5 (Fig. 1 a) was constructed from the CT images and remodeled by using the engineering simulation and 3D design software (ANSYS Inc., Canonsburg, PA). The geometry of the vertebrae was obtained from CT scan data from a healthy man. First, we checked and measured the data including the length, width, height and the different plane angles (transverse, frontal, sagittal plane) of each vertebral body according to the data of the CT scan. To simplify model preparation, basis with L3 body, we then use the Ansys command to rotate, translate and scale to produce L1, 2, 4, 5 vertebral bodies. After creating five different vertebral bodies, these bodies were then built as a lordosis whole spine model. The model construction, material properties, and validation have been well documented in previous studies and the moduli values are listed in the Table 1 [21–23]. The intact spinal FE model was further modified with DIAM implant (Fig. 1 b). The limited decompression operation was simulated as defect models were shown in Fig. 1 (c, d). The bone from the inferior aspect of the uni- or bilateral cephalad lamina and to a minimal degree from the superior aspect of the adjacent uni- or bilateral caudal lamina was resected. The half-disc was also removed under this simulation of partial discectomy. The three-dimension FE model of DIAM was modeled according to a real product with 8 mm in size with material properties obtained from the study conducted by Bellini et al. [17]. The DIAM was built and included into the FE model of the defect segment at the L3-L4 level. According to the surgical procedures, the interspinous ligament at the instrumented level was removed. A bonded contact element was assumed at the bone/implant interface. The size of mesh of the instrument compared with the laminal bony part of the spine is controlled within 1 mm. Four models were designed in this study: (1) a model of the intact spine without any implants (the INT model), (2) a defect model of the spine with bilateral laminotomies and partial discectomy at L3–4 (the DEF model), (3) a model of the spine with unilateral laminotomy and partial discectomy with the DIAM implanted at L3–4 for dynamic fixation (the DIAMUNI model), (4) a model of the spine with bilateral laminotomies and partial discectomy with the DIAM implanted at L3-L4 for dynamic fixation (the DIAMBIL model) (Fig. 1 (c, d)). The interfaces between facet articular surfaces were treated as standard contact pairs at all levels. This study aimed to investigate the biomechanical effects on the motion of segments adjacent to the level (L2-L3 and L4-L5 for the DIAMUNI and DIAMBIL models) which had undergone decompression and was implanted with the DIAM; thus, the interfaces between the DIAM and spinal process were treated as bonded. The upper (L3) and lower (L4) pars interarticularis at the DIAM implantation level (L3-L4 for the DIAMUNI and DIAMBIL models), were also investigated for comparison with the corresponding pars interarticularis of the INT and DEF models.

**Table 1:** Material properties of lumbar spine

Material	Element Type	Young’s Modulus (MPa)	Poisson’s Ratio	Area (mm2)
Bone				
Cortical	8-node SOLID185	Ex = 11,300	ν xy = 0.484	–
		Ey = 11,300	ν xz = 0.203	–
		Ez = 22,000	ν yz = 0.203	
		Gx = 3800		
		Gy = 5400		
		Gz = 5400		
Cancellous	8-node SOLID185	Ex = 140	ν xy = 0.45	–
		Ey = 140	ν xz = 0.315	
		Ez = 200	ν yz = 0.315	
		Gx = 48.3		
		Gy = 48.3		
		Gz = 48.3		
Posterior element	8-nodeSOLID185	3500	0.25	–
Disc				
Nucleus pulposus	8-node SOLID185	1.66	0.499	–
Ground Substance	8-node SOLID185	5.36	0.45	–
		C_10_ = 0.42	C_01_ = 0.105	
Annulus Fibers	2-node LINK10			
Outermost		550	–	0.76
Second		495	–	0.5928
Third		412.5	–	0.4712
Innermost		357.5	–	0.3572
Endplate	8-node SOLID185	24	0.4	–
Ligament	2-node LINK10			
ALL		7.8	–	24
PLL		10	–	14.4
TL		10	–	3.6
LF		15	–	40
ISL		10	–	26
SSL		8	–	23
CL		7.5	–	30

C_10_, C_01_ indicated two parameters of Mooney-Rivlin hyperelastic formation; d, material incompressibility parameter; *ALL* anterior longitudinal ligament, *CL* capsular ligament, *ISL* interspinous ligament, *LF* ligamentum flavum, *PLL* posterior longitudinal ligament, *SSL* supraspinous ligament, *TL* transverse ligament

### Boundary and loading condition

The lumbar spine FEMs were fixed at the bottom of the fifth vertebrae. The models were evaluated the effects on the adjacent spinal level by using the hybrid method demonstrated by Panjabi [20]. There were two steps for the application of loads to the models. An axial load of 150 N was applied perpendicular to the top of the L1 vertebrae is the first load step. In the second load step, a pure unconstrained moment was applied to ensure that the resultant range of motion (ROM) (L1-L5) for all the FEMs would be equal to the ROM corresponding to 16 degrees in flexion, 9 degrees in extension, 8 degrees in left torsion, and 6 degrees in left lateral bending. The torques applied to the models were those torques needed to match above specific ranges of motion for every model. The resultant ROM at the level adjacent to the instrumentation, the instrumented level, and the total lumbar spine levels for each model are listed in Table 2. In this study, data of the ROM at each motion segment, the maximal disc stresses at L2-L3 and L3-L4, and the facet contact forces (FCFs) of L2-L5 under flexion, extension, left torsion, and left lateral bending for all four models were gathered by the FEM software. The data have been collected from one node and these results are presented in Tables 2, 3, and 4 as percentages (DEF or DIAMUNI or DIAMBIL/INT) X 100%.

**Table 2:** ROM values of the four FEMs at all motion segments

Motion	Model	L1-L2 (Degree)	L2-L3 (Degree)	L3-L4 (Degree)	L4-L5 (Degree)
Flexion	INT	3.49 (100%)	3.72 (100%)	3.72 (100%)	4.65 (100%)
	DEF	3.49 (100%)	3.68 (99%)	4.47 (120%)	4.60 (99%)
	DIAMUNI	3.49 (100%)	3.69 (99%)	4.11 (110%)	4.59 (99%)
	DIAMBIL	3.49 (100%)	3.69 (99%)	4.11 (110%)	4.59 (99%)
Extension	INT	2.01 (100%)	1.93 (100%)	2.06 (100%)	3.05 (100%)
	DEF	1.94 (97%)	1.88 (97%)	2.54 (123%)	3.02 (99%)
	DIAMUNI	2.06 (102%)	1.97 (102%)	2.25 (109%)	3.09 (101%)
	DIAMBIL	2.10 (104%)	2.03 (105%)	2.40 (117%)	3.15 (103%)
Lateral Bending	INT	1.24 (100%)	1.39 (100%)	1.59 (100%)	2.04 (100%)
	DEF	1.32 (106%)	1.49 (107%)	1.98 (125%)	2.15 (105%)
	DIAMUNI	1.25 (101%)	1.40 (101%)	1.86 (117%)	2.04 (100%)
	DIAMBIL	1.23 (99%)	1.37 (99%)	1.84 (116%)	2.02 (99%)
Torsion	INT	1.71 (100%)	1.93 (100%)	2.22 (100%)	2.70 (100%)
	DEF	1.53 (89%)	1.78 (92%)	2.35 (106%)	2.50 (93%)
	DIAMUNI	1.62 (95%)	1.86 (96%)	2.41 (109%)	2.61 (97%)
	DIAMBIL	1.62 (95%)	1.86 (96%)	2.41 (109%)	2.60 (96%)

The percentages indicate the ROM values of all the models normalized by the corresponding ROM values of the INT model

**Table 3:** Disc stresses at cephalic adjacent levels

Motion	Model	L2-L3 (KPa)	L3-L4 (KPa)
Flexion	INT	526.09 (100%)	460.33 (100%)
	DEF	522.81 (99%)	661.07 (144%)
	DIAMUNI	522.52 (99%)	586.25 (127%)
	DIAMBIL	522.32 (99%)	586.73 (127%)
Extension	INT	286.53 (100%)	268.49 (100%)
	DEF	278.51 (97%)	355.57 (132%)
	DIAMUNI	291.74 (95%)	300.28 (112%)
	DIAMBIL	297.33 (104%)	305.06 (114%)
Lateral Bending	INT	259.94 (100%)	252.93 (100%)
	DEF	339.27 (131%)	269.63 (107%)
	DIAMUNI	223.58 (86%)	266.84 (105%)
	DIAMBIL	255.59 (98%)	306.49 (121%)
Torsion	INT	261.88 (100%)	262.09 (100%)
	DEF	228.72 (87%)	313.92 (120%)
	DIAMUNI	245.63 (94%)	324.00 (124%)
	DIAMBIL	244.96 (94%)	323.05 (123%)

The percentages indicate the disc stresses of all the models normalized by the corresponding disc stresses of the INT model

**Table 4:** Facet joint forces at instrumented levels and adjacent levels under extension

Motion	Model	L2-L3 (N)	L3-L4 (N)	L4-L5 (N)
Extension	INT	25 (100%)	35 (100%)	45 (100%)
	DEF	21 (84%)	29 (83%)	43 (96%)
	DIAMUNI	26 (104%)	24 (69%)	50 (111%)
	DIAMBIL	27 (108%)	25 (71%)	51 (113%)

The percentages indicate the facet joint forces of all the models normalized by the corresponding facet joint forces of the INT model

## Results

### ROM at instrumented level

At the instrumented level (L3-L4), the ROM values of the DEF, DIAMUNI, and DIAMBIL models were increased by 20, 10, and 10%, respectively, compared to that of the INT model during flexion and increased by 23, 9, and 17%, respectively, compared to that of the INT model during extension. The ROM values of the DEF, DIAMUNI, and DIAMBIL models were also increased during lateral bending (25, 17, 16%) and torsion (6, 9, 9%) in all three models, respectively, compared to that of the INT model, as shown in Table 2.

### Disc stress at instrumented and adjacent levels

Table 3 presents the maximum disc stress at the instrumented and adjacent levels of the INT, DEF, DIAMUNI, and DIAMBIL models. During flexion, the disc stresses of the DEF, DIAMUNI, and DIAMBIL models at the instrumented level (L3-L4) were increased by 44, 27, and 27%, respectively, compared to that of the INT model. No significant difference of disc stresses was found at the adjacent level (L2-L3). During extension, the disc stresses of the DEF, DIAMUNI, and DIAMBIL models at the instrumented level (L3-L4) were increased by 32, 12, and 14%, respectively, compared to that of the INT model. The disc stresses of the DEF, DIAMUNI, and DIAMBIL models were decreased by 3 and 5%, and increased by 4% respectively, compared to that of the INT model. During left lateral bending, the disc stresses of the DEF, DIAMUNI, and DIAMBIL models at the instrumented level (L3-L4) were increased by 7, 5, and 21%, respectively, compared to that of the INT model. At the adjacent level (L2-L3), the disc stress of the DEF model was increased by 31%, and the results of the DIAMUNI and DIAMBIL models was decreased by 14 and 2% respectively, compared to that of the INT model.

During left torsion, the disc stresses of the DEF, DIAMUNI, and DIAMBIL models at the instrumented level (L3-L4) were increased by 20, 24, and 23%, respectively, compared to that of the INT model. The DIAMBIL and DIAMUNI models exhibited lower stress at L3-L4 than the DEF model did during flexion and extension. At the adjacent level (L2-L3), the disc stresses of the DEF, DIAMUNI, and DIAMBIL models were decreased by 13, 6, and 6%, respectively, compared to that of the INT model (Table 3).

### Facet contact forces at instrumented and adjacent levels under extension

Table 4 lists the bilateral facet loads at the instrumented and adjacent levels during extension. There was no facet contact force at any adjacent facet joint during flexion. During extension, the facet joint forces of the DEF, DIAMUNI, and DIAMBIL models at the instrumented level (L3-L4) were decreased by 17, 31, and 29%, respectively, compared to that of the INT model. Furthermore, the facet joint forces of the DEF, DIAMUNI, and DIAMBIL models at the cephalic level (L2-L3) were decreased by 16%, increased by 4%, and decreased by 8%, respectively, compared to that of the INT model, while the facet joint forces of the DEF, DIAMUNI, and DIAMBIL models at the caudal level (L4-L5) were decreased by 4%, increased by 11%, and increased by 13%, respectively, compared to that of the INT model (Table 4).

### Maximum pars stresses at instrumented level

Pars stress is defined at the node in terms of Von-Mises stress on pars interarticularis. The maximum pars stresses at the instrumented upper level (L3) and lower level (L4) are listed in Table 5. During flexion, the maximum pars stresses of the DEF, DIAMUNI, and DIAMBIL models at L3 were increased by 58, 50, and 55%, respectively, compared to that of the INT model, while the maximum pars stresses of the DEF, DIAMUNI, and DIAMBIL models at L4 were decreased by 57, 14, and 14%, respectively, compared to that of the INT model. During extension, the maximum pars stresses of the DEF, DIAMUNI, and DIAMBIL models at L3 were decreased by 25, 5, and 5%, respectively, compared to that of the INT model, while the maximum pars stresses of the DEF, DIAMUNI, and DIAMBIL models at L4 were increased by 103, 48, and 60%, respectively, compared to that of the INT model. During lateral bending, the maximum pars stresses of the DEF, DIAMUNI, and DIAMBIL models at L3 were decreased by 28%, increased by 26%, and increased by 20%, respectively, compared to that of the INT model, while the maximum pars stresses of the DEF, DIAMUNI, and DIAMBIL models at L4 were decreased by 3, 29, and 29%, respectively, compared to that of the INT model.

**Table 5:** Maximum pars stresses at instrumented levels (left side)

Motion	Model	L3 (MPa)	L4 (MPa)
Flexion	INT	3.39 (100%)	2.60 (100%)
	DEF	5.34 (158%)	1.13 (43%)
	DIAMUNI	5.08 (150%)	2.24 (86%)
	DIAMBIL	5.26 (155%)	2.23 (86%)
Extension	INT	6.52 (100%)	8.17 (100%)
	DEF	4.86 (75%)	16.60 (203%)
	DIAMUNI	6.19 (95%)	12.10 (148%)
	DIAMBIL	6.14 (94%)	13.10 (160%)
Lateral Bending	INT	0.50 (100%)	1.26 (100%)
	DEF	0.36 (72%)	1.22 (97%)
	DIAMUNI	0.63 (126%)	0.89 (71%)
	DIAMBIL	0.60 (120%)	0.89 (71%)
Torsion	INT	10.90 (100%)	9.97 (100%)
	DEF	8.94 (82%)	12.20 (122%)
	DIAMUNI	9.64 (88%)	13.60 (136%)
	DIAMBIL	9.55 (88%)	13.60 (136%)

The percentages indicate the maximum pars stresses of all the models normalized by the corresponding maximum pars stresses of the INT model

**Fig. 2:**
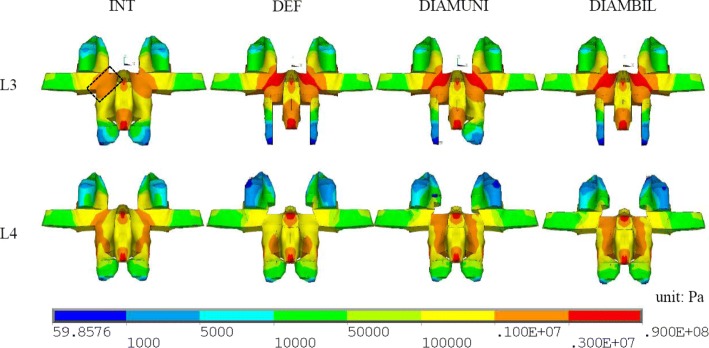
Von Mises stress distribution at the posterior lumbar lamina of L3 (above) and L4 (below) during flexion. Left Pars interarticularis (dotted box)

**Fig. 3:**
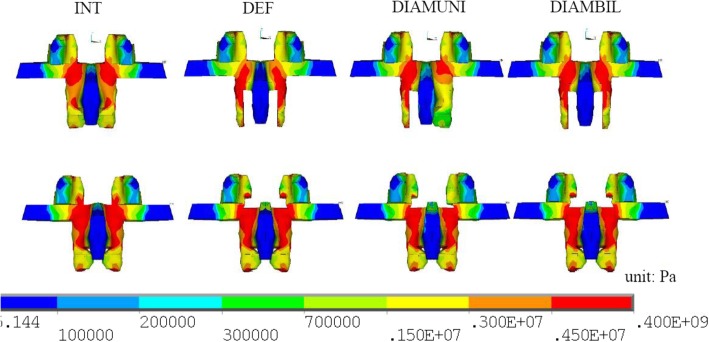
Von Mises stress distribution at the posterior lumbar lamina of L3 (above) and L4 (below) during extension

During torsion, the maximum pars stresses of the DEF, DIAMUNI, and DIAMBIL models at L3 were decreased by 18, 12, and 12%, respectively, compared to that of the INT model, while the maximum pars stresses of the DEF, DIAMUNI, and DIAMBIL models at L4 were increased by 22, 36, and 36%, respectively, compared to that of the INT model. The DEF model exhibited higher stress at the L3 pars interarticularis during flexion, but there were no significantly decreased results with either DIAM implantation model. However, the DIAM models both showed a nearly two-fold increase over the maximum pars stress of the DEF model at L4 during flexion (Fig. 2). The observed stresses at the L4 pars interarticularis were also higher during extension in the DEF, DIAMUNI, and DIAMBIL models that in the INT model. In addition, increased stresses during extension at L3 were also seen in the two DIAM models in comparison with the DEF model (Fig. 3). Such increased stresses at L3 were also observed during lateral bending in the two DIAM models in comparison with the DEF model.

## Discussion

Although conventional wide laminectomy is the standard option for lumbar structure decompression, it also causes spinal instability, and this iatrogenic spinal instability may require surgical intervention for stabilization over long-term follow-up [24, 25]. Due to the advantages of limited decompressive procedures for LSS, such as a less extensive wound area and earlier recovery, MI laminotomies are broadly applied. While the midline structures are not removed in laminotomy procedures, they have been shown to nonetheless be clinically effective in the treatment of LSS [26, 27]. In clinical practice without special instrumentation, bilateral laminotomies are likely to reduce technical difficulties and prevent perioperative complications including incidental durotomy, increased radicular deficit, and epidural hematoma. The procedure involves preserving the spinous process, the interspinous ligaments, and the facet capsules by only removing the bilateral half of the inferior part of the upper lamina and a limited amount of the superior part of the lower lamina, along with the adjacent ligamentum flavum, in the treated segment. Enlarging the space by destructing the lamina can still alter the biomechanical behavior. Biomechanical comparisons of such approaches have been conducted via in vitro [25, 27–29] and numerical studies [1, 30, 31]. The present study, meanwhile, examined the mechanical effects of DIAM-augmented lumbar surgery with laminotomy and discectomy.

The current study showed that following bilateral laminotomies with partial discectomy at L3-L4, the ROM was increased for all types of motions at the motion segment. Specifically, there were increases of 20% for flexion, 23% for extension, 25% for lateral bending, and 6% for torsion in comparison to the INT model. These findings were consistent with those of previous biomechanical investigations that found significantly increased motion at the decompressive lumbar segment after partial discectomy [32, 33]. To avoid effects such as mechanical back pain resulting from the altered stresses that can occur after the decompression procedure, the DIAM was implanted to provide stabilization for any resulting instability. The biomechanical stability was assessed by comparing the ROM at the L3-L4 level for each type of motion to the corresponding motion of the intact L3-L4 segment. The DIAM implantation was effective in reducing the ROM for all the types of motion, with the exception of torsion, after the decompressive procedures. The ROM were reduced after DIAM insertion by 10% in both DIAM models (DIAMUNI and DIAMBIL) during flexion, by 14% in DIAMUNI and 6% in DIAMBIL during extension, and by 8% in DIAMUNI and 9% in DIAMBIL during lateral bending, compared with the DEF model. During torsion, the DIAM models did not exhibit reduced ROM but rather exhibited slightly increased ROM (by 3%) compared to the DEF model. Phillips et al. [13] considered the lack of ability to control the torsion to result from the location and distraction effect of the DIAM. The DIAM is placed close to the torsional axis of rotation after decompressive procedures. The DIAM also provides a distraction force between the facet joints that is essential for controlling torsional motion and causes ineffectiveness of the facet joints by causing resistance at the axial torsional moments. In this study, the ROM at the adjacent levels was restored to close to the level of the intact segment in the DIAM models.

The measure of intradiscal pressure varies depending on the position of the transducer in the in vitro biomechanical tests, not to mention the result after partial discectomy. We were able to show that the disc stress was decreased during flexion and extension at the instrumented level with implantation of the DIAM. More specifically, the DIAM appears to redirect the load away from the residual disc during flexion and extension. During lateral bending and torsion, meanwhile, no significant decrease in disc stress was observed at the instrumented level, which suggested that the DIAM did not alter the mechanics during these two motions. Most of the pressure changes were observed in the anterior and posterior annulus. A previous study found that the lowest compressive stresses in the nucleus and anterior annulus occur in the neutral posture and that reduced stress occurs in the posterior annulus when the motion segment is positioned in extension; the authors of that study attributed this observation to the facet joints [34]. In their view, the facet joints acted as a fulcrum and redirected most of the force away from the respective disc. In this study, the DIAM effectively worked instead of the facet joints to transfer the load from the disc to the posterior element. Another focus of the study was on understanding the disc stress at the level adjacent to the implant because such information could be helpful in determining how changes in stress at that level may lead to long-term disc degeneration. Swanson et al. [35] previously reported that an IPD does not significantly change the disc stress at the adjacent levels, and our results also showed that the implant did not increase the disc stress at cephalic adjacent level. It appears, therefore, that such an implant would not induce degenerative changes at the adjacent levels, and that it may have some benefit with respect to stress-related discogenic back pain.

According to several previous studies, the highest contact force experienced by the facet joints occurred during extension [36, 37]. So we only quantified the facet force during extension, while loading during lateral bending or torsion was not addressed. The reduction in facet loads in the DEF model was probably due to the decrease in posterior laminal bony support connected to the bilateral facets. The loss of bony support resulted in a decrease in the stiffness and the loads carried by the facets. The data of the current study indicated that the DIAM did unload the facet joint stress at the implanted level, and these results were consistent with the prediction of Minns and Walsh’s study that insertion of an IPD would decrease facet joint stress at the implanted level [38]. Although those authors did not address the facet joints force at adjacent levels, our results suggested that the DIAM redirected the facet joint loading to the adjacent levels according to the increased facet joint stresses at the adjacent levels during extension. In fact, few studies have conducted direct measurements of the facet joints using pressure-sensitive film in biomechanical tests. These technique differences make it difficult to make direct comparisons with previous biomechanical studies. The IPD had the effect of decompression on the facet joint at the implanted levels, and this indicated that the IPD may be effective in treating facet-induced lower back pain.

Based on the biomechanics and anatomy of the lumbar spine, shear force is concentrated on a posterior element of the spinal column, the pars interarticularis. So the pars interarticularis is commonly the location of stress-related lesions, with stress-induced injuries commonly occurring after repetitive extension, flexion, and extension combined with rotation. Subsequent instability is a known complication due to excessive lumbar decompression over the posterior element. Ivanov et al. [10] observed increases in stresses at both the pars interarticularis and the inferior facet after limited decompressions. In this study, the DIAM models exhibited unloading of the stress by the DIAM at the pars interarticularis at L4 during extension, but increased stress at the pars interarticularis during flexion and torsion at L4 and during extension, lateral bending, and torsion at L3 compared with the DEF model. During extension, the stress value of the DEF model at the pars interarticularis at L4 was twice that of the INT model. During flexion, the DIAM models did not exhibit significant changes in stress at L3, while exhibiting increased stress at L4. The pars stress is the resultant vector of shear forces, and the bending moment comes from the articular processes. During flexion, the bending moment carried by the inferior articular process of L2 did not produce a significantly decreased effect on the pars interarticularis with the DIAM implanted between L3 and L4. However, during flexion in L4, the DIAM produced a distraction force between L3 and L4, and this force attempted to pull the facet joints away from each other and increased the shear force at the pars interarticularis in L4. During extension, the contact force produced by the inferior articular process of L3 is the major stress at the pars interarticularis of L4. The distraction effect of the DIAM decreased this facet joint force and decreased the pars stress at L4. In L3 during extension, the distraction effect from the DIAM increased the bending upward moment applied directly to the L3 and increased the L2-L3 facet contact force at the pars interarticularis in L3. This effect also was examined through our results regarding the facet joint force between L2 and L3 in the DIAM implantation models. In this study, increased stresses were found at the pars interarticularis with the IPD when compared to the DEF model during different motions. Green et al. [39] reported on alternating flexion and extension movements causing large stresses at the pars interarticularis using a human cadaveric lumbar spine. Schulitz and Niethard [40] also said that particular strains at the pars interarticularis occur through hyperextension, axial stress, and torsion of the lumbar spine. In the present study, although the pars stresses of implanted models during extension and torsion in L3 and during flexion in L4 were still lower than the stresses estimated for the INT model. The stress distribution at the pars interarticularis was still higher than the other vertebrae site. These findings suggested the pars interarticularis is a weak anatomical structure even with IPD after decompression surgery.

The increasing use of IPDs combined with limited lumbar surgical decompression has caused confusion regarding the contribution of the IPDs. According to the results of the current study, the IPD yielded stress absorptive action that decreased the ROM and unloaded the intradiscal stress and facet joint force in the implanted segment after surgical lumbar decompression. Furthermore, the IPD did alter loading condition at the implanted and adjacent levels. The pars interarticularis was considered the weak point after the limited decompressive procedure, and the IPD changed the stress distribution under different motions in the implanted segment. The surgeon should be aware of the risk of stress concentration-induced fracture when using an IPD after a limited lumbar decompression operation.

The FEM used in this study presented some limitations. The main limitation of the current study is that the model was validated against range of motion data from cadaveric studies, which also represents an indirect validation of stress estimates. However, facet contact force predictions were not validated with experimental data. Only one validated generic model was used. Inter-subject variability (anatomical, constitutive properties) was not accounted for and could affect the results of this study. Additionally, while the MI decompression is performed on the elderly population, and in most cases, other age-associated diseases as osteophytes, osteoporosis, and spine deformity. These age-related characteristics such as dehydration, reduced disc height, and facet osteoarthritic changes, were all not taken into account. These issues affect the outcomes of implantation and necessitate patient-specific finite element models to account for such factors. Thus, our INT model was not consistent with a stenosis model. Also, the two laces used to secure the DIAM in place were ignored. Because the study aimed to examine how the biomechanical effects of the DIAM work under decompression rather than to evaluate the role of the laces, the study assumed that the supraspinous ligament is able to provide enough stability to the device. These limitations should be kept in mind with regard to the conclusions drawn by this study.

## Conclusions

According to the results of this study, the disruption of the lamina and the partial disc would lead to increases in the ROM and disc stress, as well as decreased stiffness, at the treated level after a decompressive procedure. When used as a dynamic stabilizer after an MI decompression surgery, however, the DIAM provides a distraction force and unloads the disc stress and facet force in the posterior element. The study results demonstrated that one of the critical areas in the posterior region of the lumbar spine is the pars interarticularis. However, the implantation of the DIAM may not be very helpful in decreasing the stress concentrated at the pars area. Analysis of the stresses at the pars interarticularis during four motions in the four surgical models indicated that the DIAM models exhibited unloading of the stress at the pars interarticularis in L4 during extension but increased stress at the pars interarticularis during flexion and torsion in L4, as well as during extension, lateral bending, and torsion in L3, compared with the DEF model.

## Abbreviations

DEF model: Model of the spine with bilateral laminotomies and partial discectomy
DIAM: Device for Intervertebral Assisted Motion
DIAMBIL model: Model of the spine with bilateral laminotomies and partial discectomy with DIAM implantation
DIAMUNI model: Model of the spine with unilateral laminotomy and partial discectomy with DIAM implantation
FCFs: Facet contact forces
FEM: Finite element model
INT: Model of the intact spine without any implants
IPD: Interspinous process device
LSS: Lumbar spinal stenosis
MI: Minimally invasive
N: Newton
ROM: Range of motion
